
JOURNAL INFORMATION
==============================
NLM Title Abbreviation: Biospektrum (Heidelb)
Journal ID: Biospektrum (Heidelb)
NLM Journal ID: 9615685

Biospektrum

ISSN: 0947-0867
EISSN: 1868-6249

ARTICLE INFORMATION
==============================
PMCID: PMC8852920
PMID: 35194333
DOI: 10.1007/s12268-022-1706-9
Article ID: 1706
Article version: 1
Subjects: Wissenschaft Special

Kurzübersicht des SARS-Coronavirus-2-Vermehrungszyklus

Bartenschlager Ralf ralf.bartenschlager@med.uni-heidelberg.de
1 2
1 grid.7700.0 0000 0001 2190 4373 Zentrum für Infektiologie Molekulare Virologie, Universität Heidelberg, Im Neuenheimer Feld 344, D-69120 Heidelberg, Deutschland
2 grid.7497.d 0000 0004 0492 0584 Abteilung „Virus-Assoziierte Karzinogenese“, Deutsches Krebsforschungszentrum (DKFZ), Im Neuenheimer Feld 242, D-69120 Heidelberg, Deutschland
Electronic publication date: 2022 Feb 13
Print publication date: 2022
Volume: 28
Issue: 1
First page: 47
Last page: 49
Copyright: © Der Autor 2022
License: Dieser Artikel wird unter der Creative Commons Namensnennung 4.0 International Lizenz veröffentlicht, welche die Nutzung, Vervielfältigung, Bearbeitung, Verbreitung und Wiedergabe in jeglichem Medium und Format erlaubt, sofern Sie den/die ursprünglichen Autor(en) und die Quelle ordnungsgemäß nennen, einen Link zur Creative Commons Lizenz beifügen und angeben, ob Änderungen vorgenommen wurden. Die in diesem Artikel enthaltenen Bilder und sonstiges Drittmaterial unterliegen ebenfalls der genannten Creative Commons Lizenz, sofern sich aus der Abbildungslegende nichts anderes ergibt. Sofern das betreffende Material nicht unter der genannten Creative Commons Lizenz steht und die betreffende Handlung nicht nach gesetzlichen Vorschriften erlaubt ist, ist für die oben aufgeführten Weiterverwendungen des Materials die Einwilligung des jeweiligen Rechteinhabers einzuholen. Weitere Details zur Lizenz entnehmen Sie bitte der Lizenzinformation auf http://creativecommons.org/licenses/by/4.0/deed.de.
License URL: https://creativecommons.org/licenses/by/4.0/

issue-copyright-statement: © The Author(s), under exclusive licence to Springer-Verlag GmbH Deutschland, ein Teil von Springer Nature 2022

==============================
The severe acute respiratory syndrome Coronavirus type 2 (SARS-CoV-2) has caused a pandemic with major impact on human society, the economy, and our daily life. SARS-CoV-2 is a plus-strand RNA virus causing death of infected cells and an inflammation-dominated immune response. Replication of the virus occurs in the cytoplasm in distinct membranous compartments designated replication organelles, providing a shielded environment for synthesis of viral RNAs. Here, I will briefly summarize key aspects of the SARS-CoV-2 replication cycle.

■ Die COVID-19-Pandemie, verursacht durch das SARS-CoV-2, hat globale Auswirkungen auf die menschliche Gesellschaft, die Wirtschaft und das tägliche Leben. Das Virus ist höchst wahrscheinlich zoonotischen Ursprungs, d. h. es kommt natürlicherweise in einer Tierspezies (vermutlich Fledermausarten) vor und wurde auf den Menschen übertragen. Seit Ende 2019 hat sich das SARS-CoV-2 global ausgebreitet und nahezu 300 Millionen diagnostizierte Infektionen verursacht (Stand Januar 2022). Die meisten von ihnen verlaufen eher mild oder sogar asymptomatisch, dennoch sind ~5,5 Millionen Menschen an COVID-19 gestorben [1]. Die Mechanismen der Krankheitsentstehung sind nach wie vor unzureichend geklärt, resultieren aber höchstwahrscheinlich aus einem direkten zellabtötenden (zytopathischen) Effekt und einer übermäßigen Entzündungsreaktion sowie zahlreichen Begleiterscheinungen, wie etwa die Störung des Blutgerinnungssystems mit dem Risiko für Thrombosen. Die Schwere der Erkrankung wird von zahlreichen Risikofaktoren beeinflusst, wie hohes Alter der infizierten Person, vorbestehende Herz-Kreislauf-Erkrankungen oder ein hoher Body-Mass-Index (BMI).

## Viraler Vermehrungszyklus

Das SARS-CoV-2 gehört zur Familie der Coronaviridae, wozu auch das SARS-CoV, das MERS-Coronavirus sowie vier weit verbreitete „Erkältungs-Coronaviren" gehören, die ebenfalls einen zoonotischen Ursprung haben. Coronaviren sind umhüllte RNA-Viren, d. h. ihre Oberfläche besteht aus einer Lipidmembran, in die zahlreiche Kopien des Spikeproteins eingebaut sind und einem inneren Nukleokapsidkomplex, der das RNA-Genom enthält [2]. Die Infektion der Zelle wird primär durch die Bindung des Spikeproteins an den zellulären Rezeptor ACE2 vermittelt. Danach verschmilzt die Virushülle mit der Plasmamembran der Zielzelle oder das Virus wird durch die Rezeptor-vermittelte Endozytose aufgenommen. In beiden Fällen gelangt das virale Genom in das Cytoplasma, wo es von den zellulären Proteinsynthesemaschinen, den Ribosomen, abgelesen wird. Damit funktioniert das SARS-CoV-2 Genom wie eine zelluläre mRNA und wird deshalb auch als Plusstrang-RNA-Genom bezeichnet (weil mRNA *per definitionem* eine Plusstrang-Polarität hat). Die viralen Proteine erfüllen verschiedene Zwecke: Zum einen dienen die meisten Nichtstrukturproteine (*non-structural proteins*, nsp) der Herstellung viraler RNAs; zum anderen bilden die Strukturproteine, wozu das Nukleokapsid- und das Spikeprotein zählen, neue Viruspartikel, die aus der Zelle ausgeschleust werden.

## Rolle des Interferonsystems bei der Kontrolle der SARS-CoV-2-Vermehrung

Das SARS-CoV-2-Genom enthält auch die Bauanleitung für spezielle Virusfaktoren, die zelluläre Abwehrmechanismen ausschalten und somit die Virusvermehrung steigern. Unter diesen Abwehrmechanismen spielt das Interferonsystem eine zentrale Rolle, ein sehr schnell reagierendes und in quasi allen Körperzellen vorhandenes System. Dieses erkennt Eindringlinge wie das SARS-CoV-2 und setzt eine Signalkette in Gang mit zwei Ergebnissen: Erstens werden in der infizierten Zelle zahlreiche Gene aktiviert, die die Virusvermehrung ausbremsen. Zweitens produzieren die infizierten Zellen Interferon, ein Botenstoff, der in die Zellumgebung abgegeben wird, an benachbarte Zellen bindet und dort ein Genprogramm auslöst, das die Zellen gegenüber dem Virus quasi immun macht, sodass sich die Infektion nicht bzw. nur deutlich verlangsamt ausbreiten kann. Wie wichtig das Interferonsystem für die SARS-CoV-2-Abwehr ist erkennt man daran, dass Personen, bei denen Gene des Interferonsystems defekt sind, ein sehr hohes Risiko für schwere Krankheitsverläufe haben [3]. Gleiches gilt für Personen, die in einer Art Autoimmunantwort Antikörper gegen das Interferon bilden. Diese Antikörper neutralisieren das Interferon und legen damit das körpereigene Abwehrsystem lahm [4]. Auch die Beobachtung, dass Kinder wesentlich seltener schwere Krankheitsverläufe entwickeln, kann zumindest teilweise mit dem Interferonsystem erklärt werden, denn bei Kindern reagiert diese natürliche Abwehr sehr viel schneller und stärker als bei älteren Personen [5]. Und das ist wichtig, denn wenn die Abwehr sehr schnell erfolgt, hat das Virus keine Zeit, seine eigene Interferonblockade in den infizierten Zellen aufzubauen. Wenn die Interferonantwort zu langsam kommt, gewinnt das Virus und blockiert das System, sodass sich die Zelle nicht mehr gegen das Virus wehren kann und auch kein Interferon mehr abgibt, um die benachbarten Zellen zu schützen.

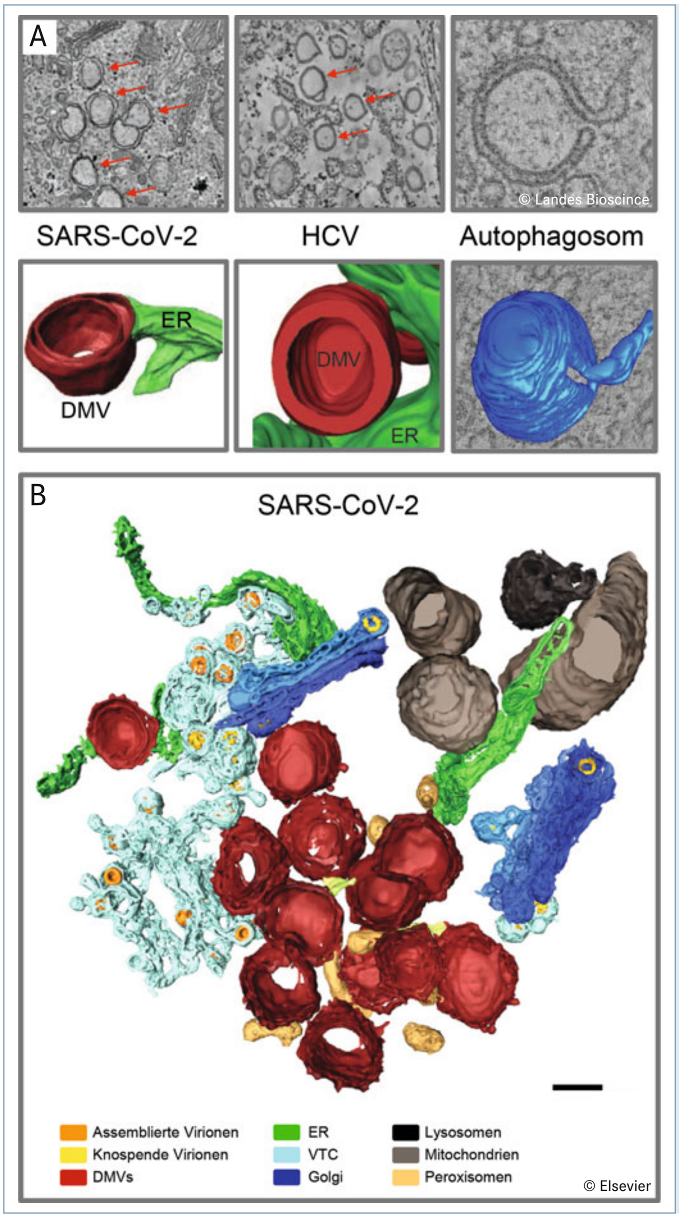

◀ **Abb. 1:** Struktur der SARS-CoV-2-Vermehrungsfabrik. **A**, Strukturvergleich SARS-CoV-2 und HCV-induzierter DMVs mit Autophagosomen. Elektronenmikroskopische Schnittbilder sind in der oberen Bildreihe gezeigt (rote Pfeile deuten auf DMVs), die entsprechenden 3D-Rekonstruktionen darunter. Abb. nach [7, 11]. **B**, 3D-Rekonstruktion einer elektronenmikroskopischen Analyse von SARS-CoV-2-induzierten DMVs (dunkelrot) und deren Anbindung an verschiedene zelluläre Membransysteme (Legende im unteren Bildteil). Abb. nach [9]. VTC: vesiculo-tubuläres Kompartiment (eine ER-abgeleitete Membranstruktur); Skalenbalken = 200 nm.

## Die SARS-CoV-2-Vermehrungsfabrik

Plusstrang-RNA-Viren vermehren sich im Cytoplasma der infizierten Zelle, wo sie spezifische Membranstrukturen hervorrufen, die sich von unterschiedlichen zellulären Membransystemen ableiten und als virale Vermehrungsfabriken (auch Replikationsorganellen (ROs) bezeichnet) dienen. Morphologisch kann man zwei RO-Typen unterscheiden: Erstens Membraneinstülpungen, beispielsweise in das endoplasmatische Retikulum (ER); dieser RO-Typ ist beispielsweise beim Dengue-Virus und dem Zikavirus zu finden [6]. Zweitens Ansammlungen von Membranvesikeln mit einer Doppelmembran; dieser RO-Typus ist etwa beim Hepatitis-C-Virus oder den Coronaviren zu finden (**Abb. 1A**). Diese Doppelmembranvesikel (DMVs) stammen primär vom ER ab und haben große Ähnlichkeit mit Autophagosomen, also den Membranen, mit denen die Zelle beispielsweise defekte Organellen umgibt, um sie anschließend abzubauen und die freigesetzten Bestandteile zu recyceln. Es ist deshalb nicht überraschend, dass das SARS-CoV-2, ebenso das Hepatitis-C-Virus und andere Plusstrang-RNA-Viren, für die Ausbildung ihrer DMVs Bestandteile der zellulären Autophagiemaschinerie nutzen [7]. Diese werden damit für die RO-Bildung zweckentfremdet, ein Paradebeispiel wie Viren die Zelle austricksen. Im Fall des SARS-CoV-2 erfolgt das primär durch die viralen Proteine nsp3 und nsp4, die Bestandteil eines sehr großen Vorläuferproteins sind, das durch zwei virale Protein-spaltende Enzyme (Proteasen) in insgesamt 16 Proteine zerlegt wird. Nur diese Spaltprodukte können ihre volle Aktivität entfalten, weshalb die Blockade der viralen Proteasen beispielsweise durch das neu entwickelte Medikament Paxlovid die Virusvermehrung lahm legt.

Unabhängig von der Morphologie der ROs haben sie eine funktionale Gemeinsamkeit: Im Innern der Membranvesikel erfolgt die Vervielfältigung des RNA-Genoms, das sich dadurch den Abwehrmechanismen der Zelle, die das RNA-Genom abbauen, entzieht. Damit die Genomvervielfältigung aber funktioniert, benötigen diese Vesikel spezielle Öffnungen, Poren, durch die Metaboliten ein- und neugebildete RNA-Genome ausgeschleust werden können. Diese Poren wurden vor kurzem für das SARS-CoV-2 beschrieben und sie werden im Wesentlichen von nsp3 gebildet [8]. Da nsp3 auch das virale Nukleokapsidprotein bindet nimmt man an, dass Virusgenome über die DMV-Pore entlassen werden und direkt an das Nukleokapsidprotein binden, um damit den Zusammenbau

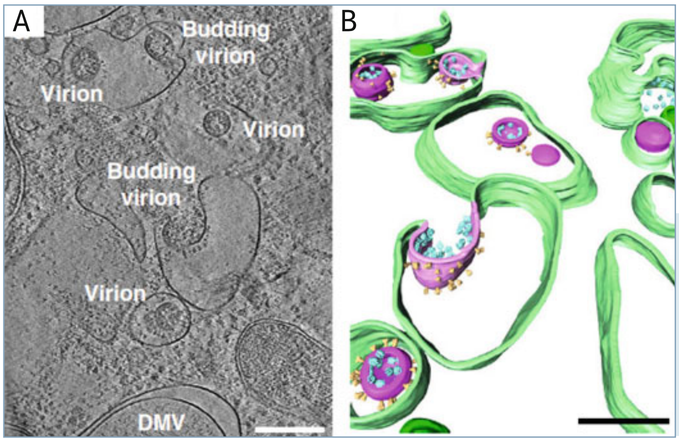

◀ **Abb. 2:** Ausbildung von SARS-CoV-2-Virionen an ER-abgeleiteten Membranen. Der linke Bildteil zeigt ein kryotomografisches Schnittbild, rechts daneben ist die 3D-Rekonstruktion dargestellt. Die zellulären und viralen Membranen sind in Grün bzw. Magenta, das Spikeprotein in Gelb und das Nukleokapsid mit dem viralen RNA-Genom in Cyan gezeigt. *Budding virion*: Viruspartikel im Prozess der Knospung. Skalenbalken = 200 nm. Abb. nach [10].

neuer Virionen einzuleiten. Diese bilden sich durch Einstülpung von zellulären Membranen, an denen sich das Spikeprotein ansammelt (**Abb. 2**). Durch einen Knospungsprozess schnüren sich Viruspartikel in das Innere bestimmter ER-Regionen ab und werden von dort über verschiedene Transportwege aus der Zelle ausgeschleust. Dabei wird das Spikeprotein durch zelluläre Proteasen in zwei Untereinheiten (S1 und S2) gespalten, was für die Infektiosität der Viruspartikel sehr wichtig ist.

## Direkt cytopathische Veränderungen

Die SARS-CoV-2-Infektion hat dramatische Folgen für die Zelle, die letztlich an der Infektion zugrunde geht. Die Ursachen hierfür sind vielfältig und nur teilweise entschlüsselt. So bringt das Virus die zelluläre Proteinsynthese in weiten Teilen zum Erliegen, weshalb wichtige zelleigene Bestandteile nicht mehr in ausreichender Menge nachgeliefert werden, während die Synthese der viralen Proteine weitgehend ungehemmt weiterläuft. Außerdem können virusinfizierte Zellen mit Nachbarzellen verschmelzen, weil das Spikeprotein auch an der Zelloberfläche zu finden ist und dort durch Bindung an das ACE2 von Nachbarzellen die Verschmelzung der Zellmembranen einleitet. Darüber hinaus verursacht SARS-CoV-2 eine massive Veränderung des zellinneren Gerüstsystems (das Cytoskelett), was sehr wahrscheinlich zu Störungen intrazellulärer Transportvorgänge führt [9]. Diese wenigen Beispiele belegen die vielfältigen Störungen von SARS-CoV-2 in der infizierten Zelle.

**Korrespondenzadresse:**

Prof. Dr. Ralf Bartenschlager
Zentrum für Infektiologie
Molekulare Virologie
Universität Heidelberg
Im Neuenheimer Feld 344
D-69120 Heidelberg

Abteilung „Virus-Assoziierte Karzinogenese“
Deutsches Krebsforschungszentrum (DKFZ)
Im Neuenheimer Feld 242
D-69120 Heidelberg
ralf.bartenschlager@med.uni-heidelberg.de

Hier steht eine Anzeige.

Springer

AUTOR

**Ralf Bartenschlager**


Funding note

Open Access funding enabled and organized by Projekt DEAL.

Ralf Bartenschlager Biologiestudium und Promotion an der Universität Heidelberg. 1991–1993 Postdoktorand bei der Hoffmann-La Roche AG, Basel. 1994–2002 Arbeitsgruppenleiter am Institut für Virologie, Universität Mainz; dort ab 2000 Professor für Molekulare Virologie. 2003 Wechsel auf die Chica-und-Heinz-Schaller-Stiftungsprofessur für Molekulare Virologie, Universität Heidelberg. Seit 2014 zusätzlich Leiter der Abteilung Virus-assoziierte Karzinogenese, DKFZ.


Literatur

[1] https://coronavirus.jhu.edu/map.html
[2] V’kovski P Kratzel A Steiner S Coronavirus biology and replication: implications for SARS-CoV-2 Nat Rev Microbiol 2021 19 155 170 10.1038/s41579-020-00468-6 33116300 PMC7592455
[3] Zhang Q Bastard P Liu Z Inborn errors of type I IFN immunity in patients with life-threatening COVID-19 Science 2020 370 eabd4570 10.1126/science.abd4570 32972995 PMC7857407
[4] Bastard P Gervais A Le Voyer T Autoantibodies neutralizing type I IFNs are present in ∼4% of uninfected individuals over 70 years old and account for ∼20% of COVID-19 deaths Sci Immunol 2021 6 eabl4340 10.1126/sciimmunol.abl4340 34413139 PMC8521484
[5] Loske J, Röhmel J, Lukassen S et al. (2021) Pre-activated antiviral innate immunity in the upper airways controls early SARS-CoV-2 infection in children. Nat Biotechnol 10.1038/s41587-021-01037-910.1038/s41587-021-01037-9 34408314
[6] Welsch S Miller S Romero-Brey I Composition and three-dimensional architecture of the dengue virus replication and assembly sites Cell Host Microbe 2009 5 365 375 10.1016/j.chom.2009.03.007 19380115 PMC7103389
[7] Twu WI Lee JI Kim H Contribution of autophagy machinery factors to HCV and SARS-CoV-2 replication organelle formation Cell Reports 2021 37 110049 10.1016/j.celrep.2021.110049 34788596 PMC8577994
[8] Wolff G Limpens RWAL Zevenhoven-Dobbe JC A molecular pore spans the double membrane of the coronavirus replication organelle Science 2020 369 1395 1398 10.1126/science.abd3629 32763915 PMC7665310
[9] Cortese M Lee JY Cerika B Integrative imaging reveals SARS-CoV-2-induced reshaping of sub-cellular morphologies Cell Host Microbe 2020 6 853 866.e5 10.1016/j.chom.2020.11.003 PMC7670925 33245857
[10] Klein S Cortese M Winter SL SARS-CoV-2 structure and replication characterized by in situ cryo-electron tomography Nat Commun 2020 11 5885 10.1038/s41467-020-19619-7 33208793 PMC7676268
[11] Kondylis V van Nispen Tot Pannerden HE van Dijk S Endosome-mediated autophagy: an unconventional MIIC-driven autophagic pathway operational in dendritic cells Autophagy 2013 9 861 880 10.4161/auto.24111 23481895 PMC3672296
